# The quality monitoring of paracetamol medicament using a noninvasive microwave sensor

**DOI:** 10.1038/s41598-023-43409-y

**Published:** 2023-10-14

**Authors:** Youness Zaarour, Fatimazahrae EL Arroud, Hafid Griguer, Rafiq El Alami, Mohammed El Kohen, Wiam Salhi, Abdessamad Faik, M’hamed Drissi

**Affiliations:** 1 Microwave Energy Sensing (MSE), DICE-University of Mohammed VI Polytechnic, 43152 Benguerir, Morocco; 2 Laboratory for Inorganic Materiels for Sustainable Energy Technologies (LIMSET), University of Mohammed VI Polytechnic, 43152 Benguerir, Morocco; 3 Univ Rennes, INSA Rennes, IETR, UMR CNRS 6164, 35000 Rennes, France

**Keywords:** Electrical and electronic engineering, Drug development

## Abstract

Environmental conditions, including temperature, humidity, and light, can impact the quality of drugs. Microwave-based approaches offer a fast and cost-effective way to detect quality variations, providing an alternative to traditional techniques in the pharmaceutical and cosmetic industries. This article proposes the use of a microwave sensor for monitoring the quality of pharmaceutical drugs at distinct temperature levels. A small planar sensor based on three hexagonal split ring resonators (TH-SRR) is fabricated. The design is manufactured on an FR-4 dielectric substrate. The sensor is tested on a 1000 mg paracetamol tablet, at temperatures ranging from 40 to 80 $^\circ$C. The Variation in the permittivity that characterizes product degradation is translated into a shift in the frequency of the scattering matrix elements. To validate the microwave approach, drug quality is examined with the laser-induced breakdown spectroscopy (LIBS) technique, an optical emission laser used for both qualitative and quantitative investigations of elements contained in a sample. The existing elements are classified using the National Institute of Standards and Technology (NIST) database and categorized according to their spectral line wavelengths. The experiments show the presence of optimal wavelength values for carbon (C), hydrogen (H), nitrogen (N), and oxygen (O) at 247.92 nm, 656.49 nm, 244.23 nm, and 777.48 nm, respectively. The microwave experimental results show a shift frequency of approximately 1 MHz on average when the tablet is heated at 80 $^\circ$C for 15 min. Meanwhile, the LIBS measurement shows a remarkable shift in terms of intensity of approximately 8884 and 812 for carbon and hydrogen, respectively. Understanding how paracetamol dries under high temperatures and improving the process settings of the microwave sensor are investigated and assessed in this work.

## Introduction

Maintaining pharmaceutical quality during manufacturing has always been a priority for the World Health Organization (WHO)^1^. Every factor, whether individually or collectively, that affects product quality falls under the broad definition of quality control. Therefore, health outcomes can be plainly at risk if these products are not assessed to meet minimum criteria for quality, safety, efficacy, and significant medical needs. Good manufacturing practices (GMP) and other features, such as product design and development, are thus incorporated into quality assurance^2,3^. According to Diener et al., paracetamol is an analgesic used to relieve pain from a headache or other discomfort, but if it is not stored properly under predetermined conditions, its use could lead to kidney failure, liver damage, or the complete collapse of other essential organs^4^.

Temperature and humidity are the most crucial factors affecting the effectiveness of medicinal formulations. The aim of stability testing is to show the quality variation of a drug product over time under the effects of various environmental conditions^5^, which can accelerate oxidation, reduction, and hydrolysis reactions that lead to drug degradation.The findings consistently revealed that increasing the temperature from 25 to 45 $^\circ$C across all storage conditions resulted in an extended disintegration time for the paracetamol tablets. These results underscore the significance of temperature as a crucial factor affecting the effectiveness and stability of medicinal formulations, emphasizing the importance of rigorous stability testing to ensure product quality and efficacy over time^6^. In addition to losing some of their effectiveness and decreasing their concentration, improperly stored drugs might even induce new or dangerous impacts. For this reason, Big Pharma (a collective term for major multinational pharmaceutical companies) is looking for effective solutions to control environmental conditions throughout the supply chain (production, storage, transport, etc.) in general but especially in tropical areas where humidity and temperature are high^7–9^. During processing, drugs are generally sampled at specific periods to measure their quality in relation to their moisture content. This method is time-consuming and only supplies an average result for the analyzed tablets^10^. Pharmaceutical quality analysis protocols are essentially based on offline and cumbersome laboratory methods. Although these methods are relatively effective and offer high-precision qualitative analysis, these so-called destructive methods remain unsuitable for online and portable analysis. In fact, Big Pharma is looking for online analysis solutions capable of providing drug quality data throughout the supply chain.

In the context of drugs and equivalent substances, many interesting technologies can be found in the literature for qualitative online analysis, such as optical and infrared spectroscopy^11^, chromatographic analysis^12^, and microwave sensing spectroscopy^13^. Microwave sensing technology has long been utilized as a fast and accurate method in research and industrial equipment, such as medical devices for glucose measurement^14–16^, gas sensing^17,18^, humidity sensing^19^, or breast tumors^20,21^. This technique may offer an effective way to monitor drug quality in real-time and consequently reduce the time required for this task. Overall, this approach involves a direct interaction between an electromagnetic (EM) field created by a sensor and the material under test (MUT) for better extraction of the required information. It operates by monitoring resonance parameters (resonant frequency, amplitude, or quality factor) for permittivity variations caused by variations in the characteristics of the substance. Various studies have described this technique, with microwave sensors used for different applications. In^22^, a miniature nondestructive sensor using a microwave microstrip ring resonator was developed to assess the relative humidity of a single grain of wheat. This method was based on a microstrip line’s electrical characteristics, which depend on the dielectric constant and thickness of the material that is being layered over it. Additionally, a microstrip-based sensor without a resonator that could be inserted directly into a container was suggested in^23^ for grain quality evaluation and process control^24^. Similarly, a test was carried out to examine the impact of microwave drying on the degradation of acetylsalicylic acid, a moisture-sensitive model medication^25^. Additionally, the impact of various microwave powers (300, 600, and 900 W) on the degradation of the medication was examined. To the best of our knowledge, we did not identify in the open literature any other drugs with online qualitative analysis solutions based on a planar microwave sensing approach, especially for solid paracetamol. The present paper proposes a design, fabrication, and experimental study of a new low-cost miniature and planar microwave sensor deployed for solid paracetamol quality analysis under specific heating conditions. It mainly focuses on developing an online real-time portable device that can track drug quality by the calculated transmitted signal of the sensor while it is propagating through the drug. The functioning of the proposed microwave sensor, which operates at 3.48 GHz, is examined using a three-hexagonal split ring resonator (TH-SRR). The primary parameter studied here is the temperature condition, which directly affects the quality of drugs transported from Big Pharma to local shops. Temperatures can be increased by 22–27 $^\circ$C in an hour and reach a maximum of 89 $^\circ$C given the right circumstances, such as no ventilation and complete sun exposure. For this reason, we are estimating 80 $^\circ$C as the highest temperature that can be achieved within a drug transport vehicle. In addition, it is necessary to avoid approaching the melting point of paracetamol, which is near 100 $^\circ$C^26,27^ With the objective of confirming the heating impact on paracetamol drug quality, we chose a quantitative experimental analysis setup based on laser-induced breakdown spectroscopy (LIBS) that has been used in the pharmaceutical industry for qualitative drug analysis^28^. This technique is capable of providing us with a spectrum that depicts several intensities. These intensities are situated at standardized wavelengths corresponding to the atomic level of each chemical element of the material under analysis.

In this work, the design rules and the main optimized structure of the sensor are presented. In addition, parametric simulation and the principal work are highlighted. Finally, the potential use of the sensor and the experimental results of both microwave and LIBS measurements are analyzed and assessed using the same heated sample of solid paracetamol.

## Material and methods

### Sample preparation and heating procedure

Paracetamol, also known as acetaminophen, is a chemical compound found in many drugs, including the most commonly prescribed by doctors. Paracetamol has long been the most widely used medication for relieving pain due to its excellent safety and tolerability record. Surprisingly, subsequent investigations have raised concerns about its therapeutic efficacy, especially under extreme environmental storage conditions. It is safe and effective when used and stored in an appropriate place but could possibly suffer from a loss of potency or even a reduction in efficacy, which could be harmful when stored at high temperatures. In our experiment, we employed solid effervescent paracetamol tablets (Doliprane)^29^ that were purchased from a local pharmacy in Benguerir (Morocco) and kept under standard storage conditions. These 1000 mg tablets were obtained from the same production batch. To avoid any contamination, sample preparation was carried out in a controlled chamber with a set temperature (27 $^\circ$C) and 30% humidity, and sample manipulation was performed while wearing protective gloves. For the analyses of temperature impacts on drug quality, several samples of heated paracetamol tablets were prepared. These drug samples were uniformly heated at 40 $^\circ$C, 60 $^\circ$C, and 80 $^\circ$C with varying lengths of time from 15 to 60 min with a step of 15 min. As the objective was to simulate the temperature variations that might occur during the transport of the drugs, the chosen temperatures did not exceed 80 $^\circ$C. The samples underwent the heating procedure described in Table 1:

- First, at a fixed temperature (80 $^\circ$C), a set of four samples S1, S2, S3, and S4 was prepared to analyze the impact of the heating period on the paracetamol tablets.
- Furthermore, at a fixed period, three additional samples (S5, S6, S7) were heated at 80 $^\circ$C to confirm the accuracy, readability, and reproducibility of the paracetamol drug behavior toward the sensor’s response.
- Additionally, sample S8 was heated to 80 $^\circ$C to track the effect of the temperature on the paracetamol tablet.
- Finally, two further samples S9 and S10 were heated at 40 $^\circ$C and 60 $^\circ$C, respectively, at a fixed heating period.

**Table 1:** The 10 prepared samples for studying the effect of the heating period and various temperatures.

Samples	Period (min)	Temperature ($^\circ$C)
S1	15	80
S2	30	80
S3	45	80
S4	60	80
S5	15	80
S6	15	80
S7	15	80
S8	15	80
S9	15	40
S10	15	60

### Microwave setup

**Figure 1:**
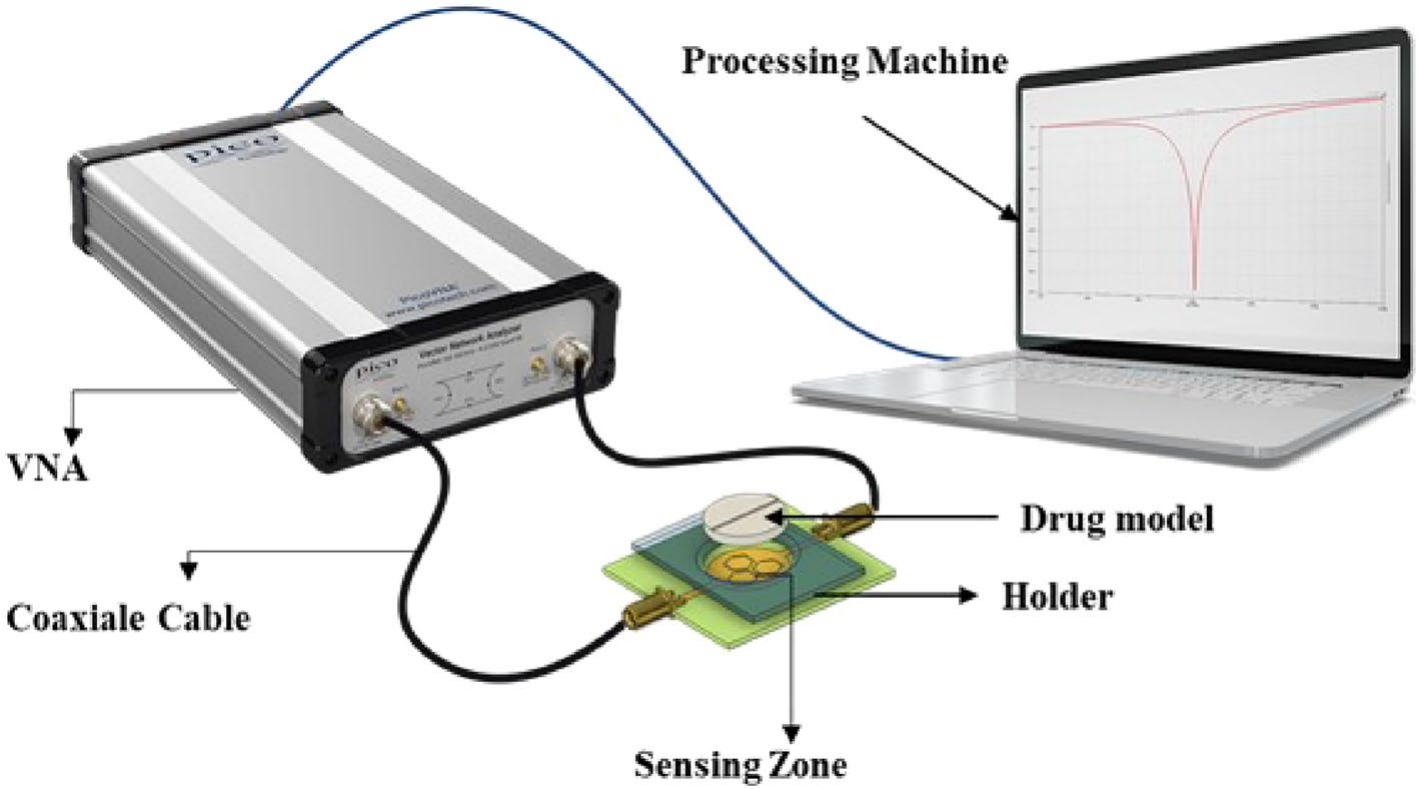
Setup used for microwave measurement.

A two-port vector network analyzer (PICO VNA 108) was employed for microwave analysis. The VNA provides 0.03 MHz of frequency resolution and 0.01 dB of magnitude resolution. A SOLT (Short, Open, Load, Through) kit was used to calibrate the VNA in a frequency range from 2 to 4 GHz with a step of 0.5 MHz. This frequency band was chosen to provide a simple method for dielectric dispersion analysis. Our choice of operating frequency bands was guided by key considerations, particularly the extensive study of dielectric properties of organic materials over 2 GHz. This range aligns with molecular processes in compounds like Paracetamol, where observable dielectric changes are induced by molecular interactions and the realignment of polar functional groups . Paracetamol’s possession of polar functional groups, such as amide and hydroxyl groups, makes it plausible to anticipate a dielectric response in this frequency band^30,31^. Our study aims to connect these dielectric property changes to molecular structure and interactions, offering valuable insights into its behavior and applications. Additionally, the chosen frequency range of 2–4 GHz aligns practically with cost-effective commercial microwave components, making sensor implementation more accessible and reinforcing our research goals.To avoid any Fairfield coupling or external noises within the electromagnetic environment surrounding the sensor, the VNA microwave power was set at − 10 dBm to cover the selected frequency band. The VNA was able to provide real and imaginary parts of the Sij {i,j: 1,2} parameter matrix. In our study, we chose the $S_{21}$ parameter, which represents the magnitude in dB of the transmission coefficient between the two VNA ports. For microwave transductance, we chose a two-port planar microwave sensor based on a TH-SRR (three hexagonal split ring resonator). The detailed design and results of the TH-SRR will be presented in the next sections. Our MUT was a 1000 mg paracetamol tablet. To ensure the proper location of the tablet above the sensor, a holder made of plexiglass with a relative permittivity of $\varepsilon _r=3.4$ was installed in this area for MUT sensing. The holder had a suitable receptacle to secure the paracetamol tablet, which was in direct contact with the upper part of the sensor above the sensing area. The diameter of the holder receptacle was 23 mm to match the tablet diameter. The two ports of the sensor were linked to the VNA with a coaxial cable matched with 50$\Omega$ impedance. Real-time processing software was launched on a laptop to receive data from the VNA via serial communication (Fig. 1).

**Figure 2:**
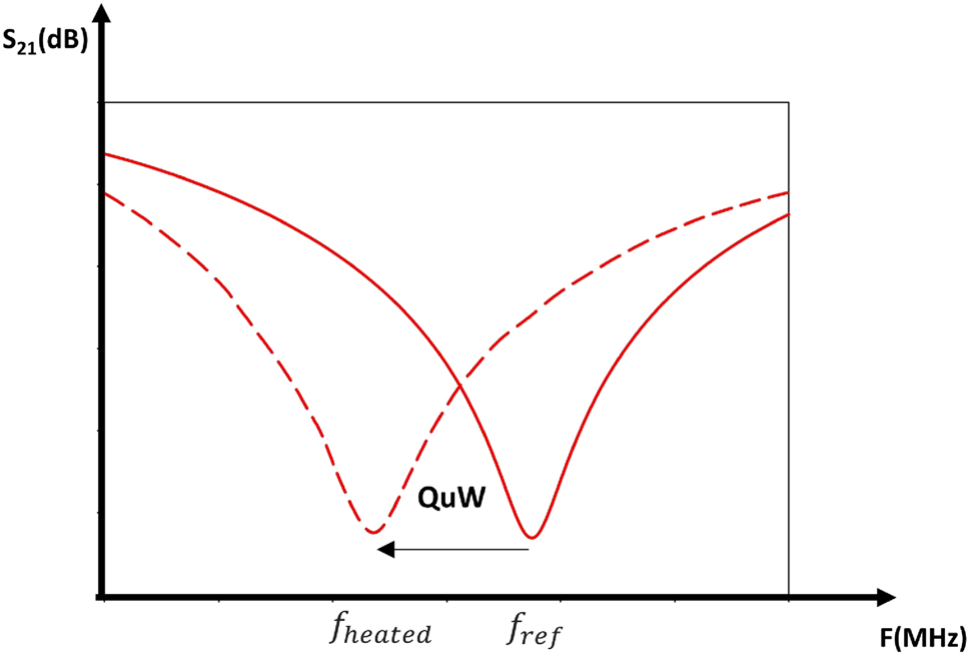
Definition of microwave quality shifting $Q_{uW}$.

The following parameter is defined to qualitatively analyze the performance of the proposed microwave sensor in different heating procedures. The microwave quality shifting $Q_{\mu W}$ is defined as:

1 $$\begin{aligned} Q_{\mu W}=f_{ref}-f_{heated} \qquad (MHz) \end{aligned}$$

Where $f_{ref}$ is the resonance frequency of the sensor loaded with a paracetamol drug at room temperature. This frequency is considered as the reference. $f_{heated}$ is the resonance frequency of the sensor for a drug heated at a specific value of temperatures (Fig. 2).

### Laser induced breakdown spectroscopy (LIBS) technique

To validate the microwave approach used for tracking the drug microwave quality shift introduced in the previous section, the LIBS technique was used to provide a reference laboratory analysis. LIBS is a sensitive optical method that can perform fast multielement analysis. It is based on establishing a qualitative and quantitative examination of the substance under test. This method of elemental analysis for pharmaceutical drug production can ultimately improve human health. In the past few years, LIBS has attracted interest from the pharmaceutical industry. This method has been utilized only in the examination of solid dosage forms for the purposes of quantifying active pharmacological ingredients in pharmaceuticals up to this point^32^. In the present research, we used the LIBS setup to identify the elements present in pharmaceutical 1000 mg paracetamol tablets. A pulsed laser was used as an excitation source in this investigation, with a wavelength of 1,064 nm and an energy expenditure of 25–50 mJ per pulse. A spectrometer is used with a wide spectral range, high sensitivity, and fast response rate with a time-gated detector. It was connected to a computer for rapid processing and interpretation of the acquired data. LIBS was employed to examine and provide an analytic spectrum with quantitative intensities for the main chemical elements forming paracetamol (C, H, N, and O) at different temperature levels. These elements were extensively analyzed to ensure that they could be identified with high accuracy and little uncertainty. The data were gathered using a laser source and spectrometer to examine the spectral lines associated with different elements according to their wavelength and energy.

### Sensor design

**Figure 3:**
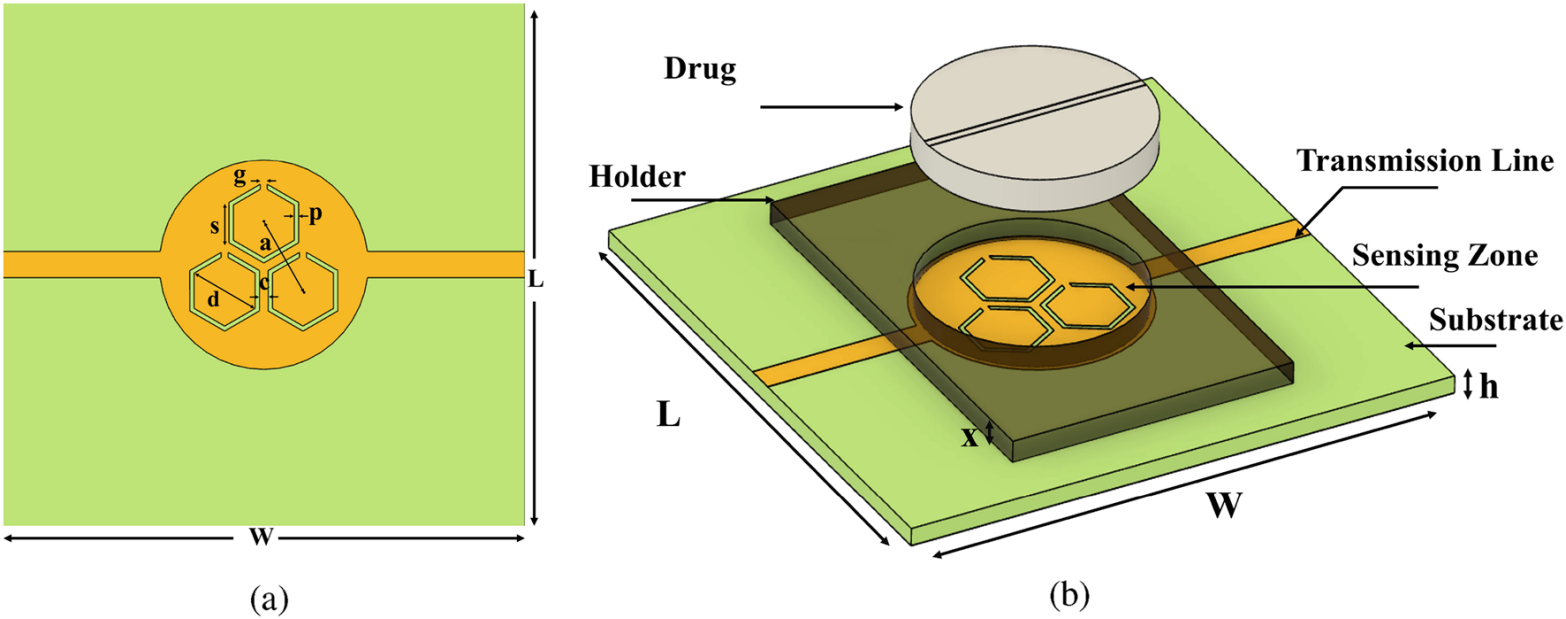
(**a**) Top view and (**b**) Cross-sectional view of the sensor. (Length L = 60 mm, width W = 60 mm, thickness h = 1.6 mm, mutual coupling distance a = 9 mm, resonator’s side s = 4.6 mm, diagonal length d = , gap g =  and path width p = 0.4 mm).

The proposed symmetrical sensor is designed to operate at approximately 3.48 GHz. The establishment of the sensor geometry followed a systematic approach to attain an optimal configuration. Initially, we opted for a circular patch identical in size to the Paracetamol tablet. This choice not only facilitated direct contact between the sensor and the drug, but also ensured surface compatibility, enabling accurate dielectric property sensing. In conjunction with the circular patch, our design incorporated three hexagonal split ring resonators (TH-SRR) excited by a double transmission line (Fig. 3a), enhancing the sensor’s overall performance. The sensor is fabricated in an FR-4 dielectric substrate with $\varepsilon _r$ = 4.4 and tan$\delta$ = 0.02, as shown in Fig. 3b. This array of hexagonal resonators is adopted to create a concentrated electromagnetic near field, which is the result of the coupling between the adjacent hexagonal edges. This EM near field is focused and oriented perpendicular to the resonator plane, thus penetrating the tablets. To achieve impedance matching, the transmission line is constructed with an 18 mm strip length and a 3 mm strip width coupled to a 50$\Omega$ coaxial SMA connection along both sides. This hexagonal resonator array remains among the best shapes to acquire a strong coupling between individual resonator edges and produce a high-sensitivity behavior to detect slight variations in the microwave properties of MUTs. A simulation was performed with a high-performance HFSS 20.1 full-wave electromagnetic EM field simulator.

**Figure 4:**
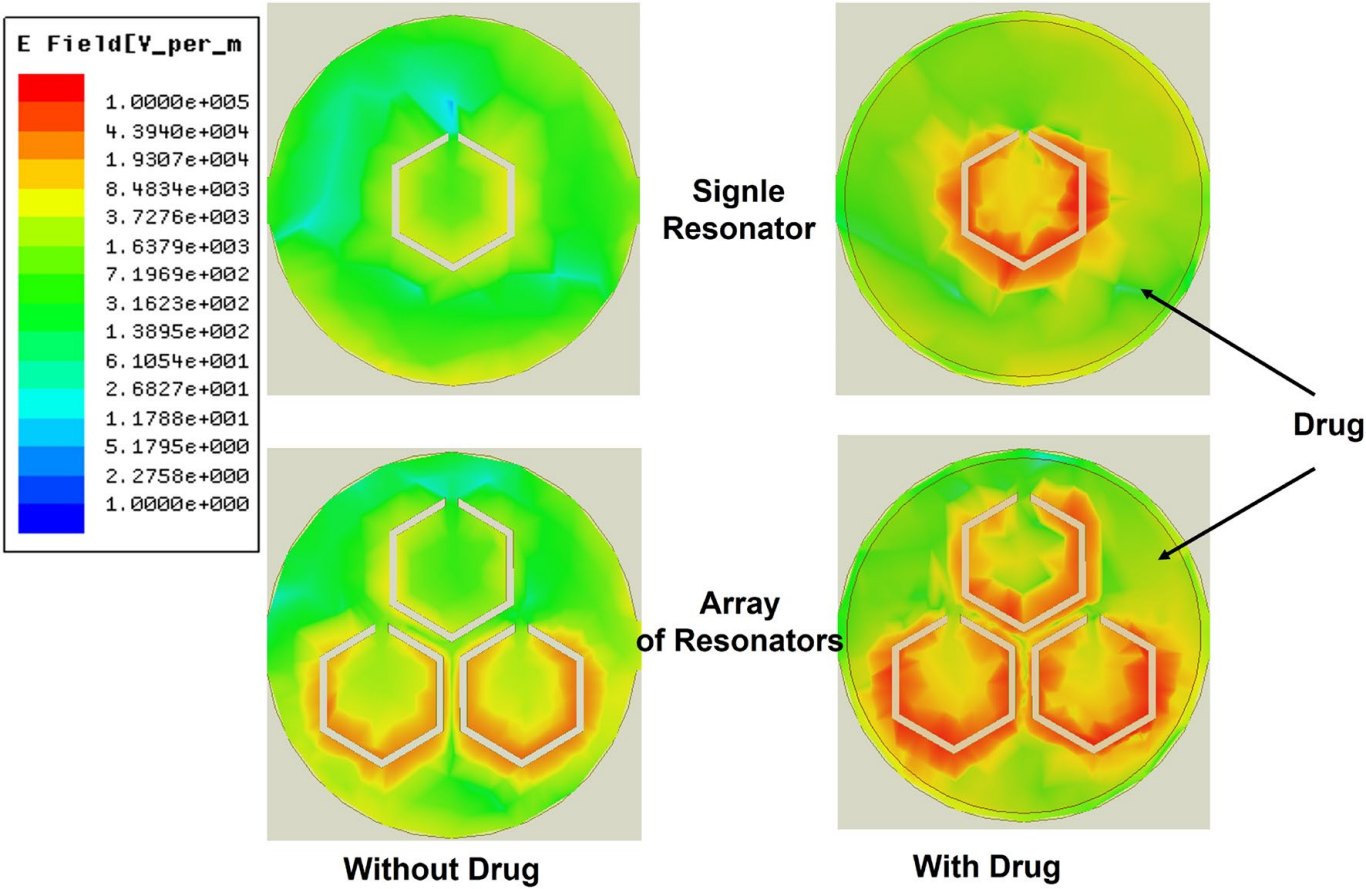
Full-wave simulation of the electric field distribution $\overrightarrow{E}$ in the upper surface plane for individual and array of resonators setups at 3.48 GHz with and without drug samples.

The arrangement of three resonators offers various possibilities to control the sensor’s response. Two of the critical factors that significantly influence the sensor’s response are the gap of the resonator and the distance separating them, also known as mutual coupling distance. These factors significantly shaped response and overall sensitivity. Through meticulous optimization, we fine-tuned these parameters, leading to the final sensor geometry. In our case, as shown in Fig. 3a, the chosen array of three resonators is crafted to form a maximum interaction between six resonator edges. All resonators are separated by a mutual coupling distance of a = 9 mm between their geometric centers. Each resonator has a side of s = 4.6 mm with a gap of g = 0.6 mm and a path width of p = 0.4 mm. The diagonal length is d = 8 mm, and the resonators are coupled to each other with c = 1 mm. As shown in Fig. 4, in comparison to a single hexagonal resonator, the TH-SRR design exhibits a greater level of electric field confinement in the 20 $\times$ 20 mm$^2$ sensing area, with an intensity reaching $10^4$ V/m around the dielectric gaps, as well as the pathways connecting them. This high intensity can be explained by the significant interaction between the highly localized fields resulting from the greater electric field in the coupling region of the TH-SRR. The small width of the slots makes the resonators extremely sensitive to variations in the characteristics of the drug under examination and improves global sensitivity.

## Results and discussion

To describe the intrinsic behavior of the unloaded sensor in depth, a parametric study using a geometric tuning process was carried out on a variety of parameters. It is known that the geometrical shape and resonator parameters define the resonance frequency of the proposed sensor when facing a specific material under test^33,34^. Additionally, determining the sensor resonance behavior depends critically on the design characteristics of each resonator and the coupling topology, such as the split gaps and the coupling distance between the resonators. The objective of this parametric study through simulation analysis is to obtain the optimal geometrical configuration that exhibits a higher sensitivity defined by a narrow frequency bandwidth and attenuated $S_{21}$ response. The bandwidth is defined here by the difference in the cutting frequencies at − 3 dB of the $S_{21}$ magnitude peak. In general, the reached array of the split ring resonators can be modeled as a shunt LC^35^, where L is the equivalent inductance formed by the mutual inductances obtained by every two adjacent resonators and C is the equivalent capacitance formed by all the split gaps of the resonator’s array.

**Figure 5:**
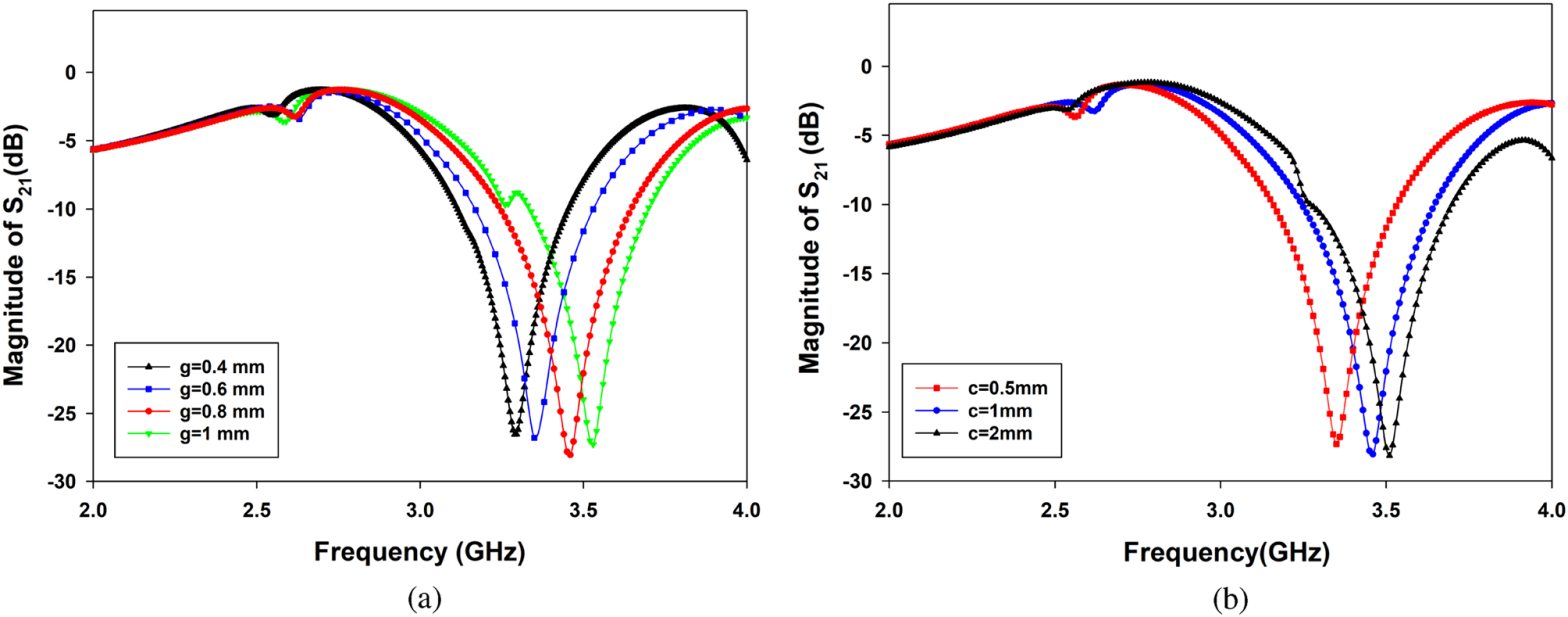
Full-wave simulation of unloaded TH-SSR sensor for (**a**) split gap variation from g = 0.4 mm to 0.8 mm, with c = 1 mm; (**b**) coupling distance variation from c = 1 mm to 2 mm, with g = 0.8 mm.

In our case, as shown in Fig. 5, the full-wave simulation shows that the unloaded sensor exhibits band gap resonance behavior at approximately 3 GHz. Furthermore, the resonance peak moves toward higher frequencies while the split gap g is changed from 0.4 to 1 mm with a step of 0.2 mm. This is because the equivalent capacitance decreases as the gap size increases, hence increasing the dependency of the transmission response (Fig. 5a). The optimal sensitivity is obtained with the g = 0.8 mm configuration, which provides a narrower bandwidth at a 3.48 GHz resonance frequency. The impact of mutual coupling distance c is examined in Fig. 5b, revealing heightened sensitivity at c = 1 mm. Notably, it is important to emphasize that beyond this point, specifically after c = 2 mm, the resonance frequency shows no substantial influence on its magnitude.

**Figure 6:**
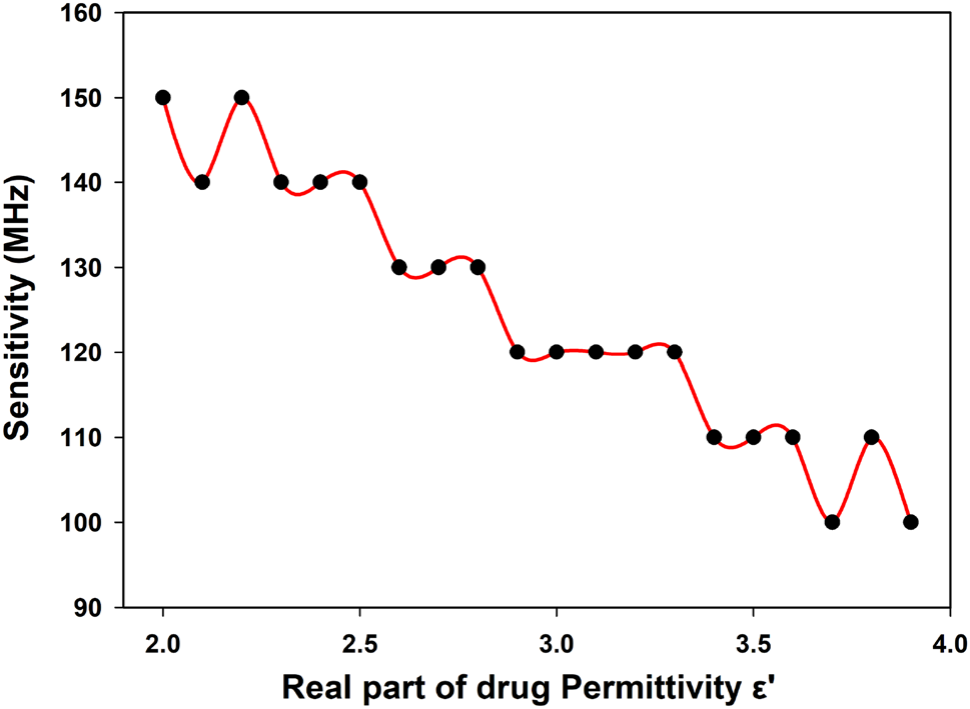
Sensitivity of the TH-SSR based sensor versus the different value of the real part of permittivity $\varepsilon ^{'}$.

After a thorough geometrical study, we proceeded to simulate and analyze the sensor’s response to changes in the dielectric properties of the drug. As part of this analysis, we modeled the drug as a material under test, varying its permittivity within the range of 2 to 4. By examining the sensor’s sensitivity across different permittivity values, we gained valuable insights into its performance characteristics and its ability to detect and measure variations in the dielectric properties of the drug. Figure 6 illustrates the sensitivity of the TH-SSR sensor based on its resonance frequency for different permittivity values ranging from 2 to 4 with a step of 0.1. The sensitivity (S) is expressed as the change in resonance frequency divided by the change in permittivity S=$\Delta f_r$/$\Delta \varepsilon _r$. The results clearly demonstrate a non-linear relationship between the sensor’s sensitivity and the permittivity value. The sensitivity is highest around a permittivity value of 2, reaching approximately 150 MHz. However, as the permittivity value increases, the sensitivity gradually decreases, reaching around 98 MHz for higher permittivity values.

**Figure 7:**
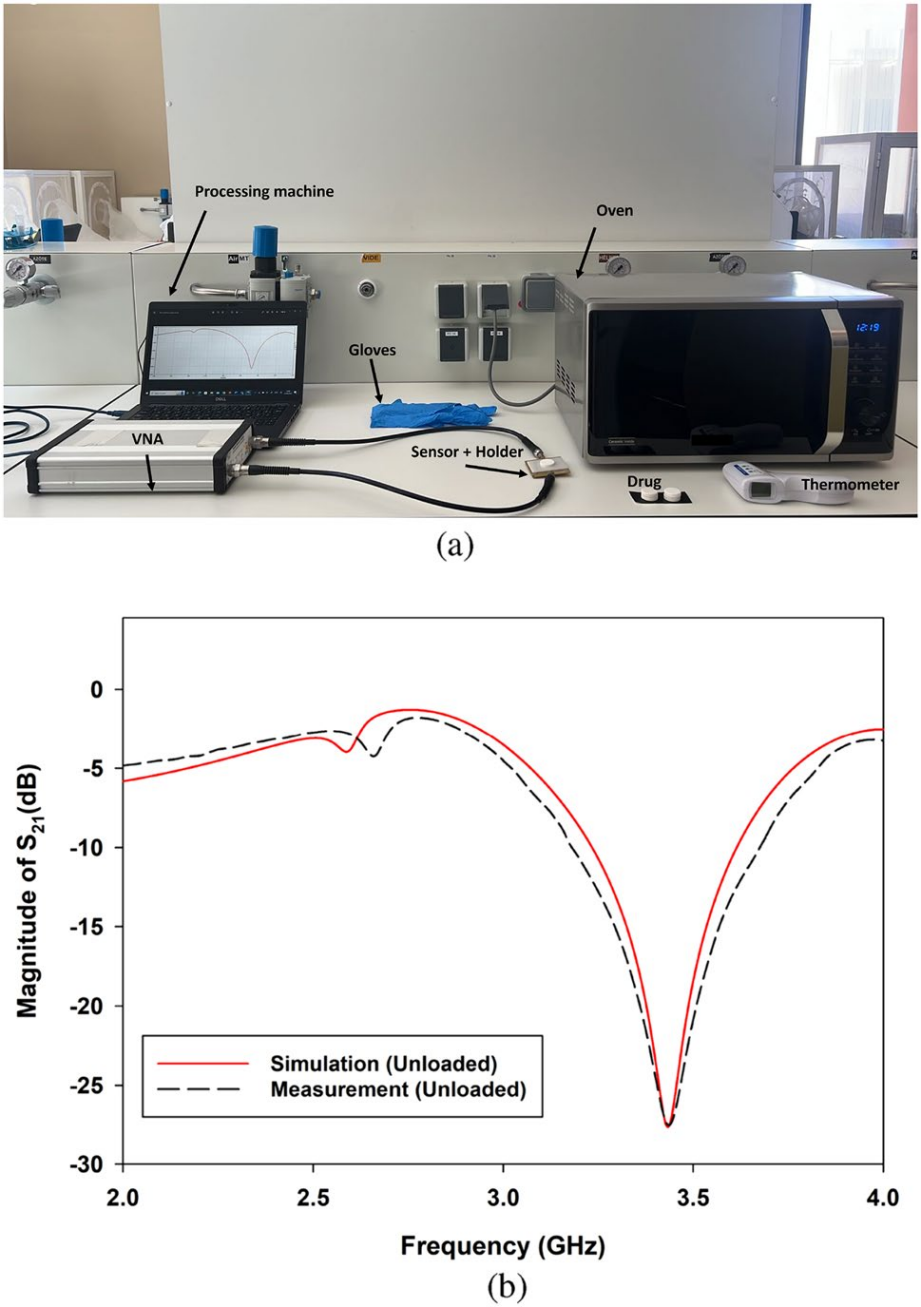
(**a**) Experimental setup, (**b**) simulated (solid line) and measured (dashed line) result of the unloaded sensor.

Following the simulations and analysis, we proceeded to experimentally evaluate the fabricated sensor using the test configuration depicted in Fig. 7a. As illustrated in Fig. 7b, the measured transmission coefficients $S_{21}$, as a function of frequency (dashed line) for the unloaded sensor and the numerical findings (continuous line), are in good agreement. The unloaded sensor’s resonance is approximately 3.48 GHz, and its resonance peak is approximately − 27.6 dB within a BW of 2 MHz. This optimized sensor design will be used in future investigations since it provides the highest sensitivity performance.

**Figure 8:**
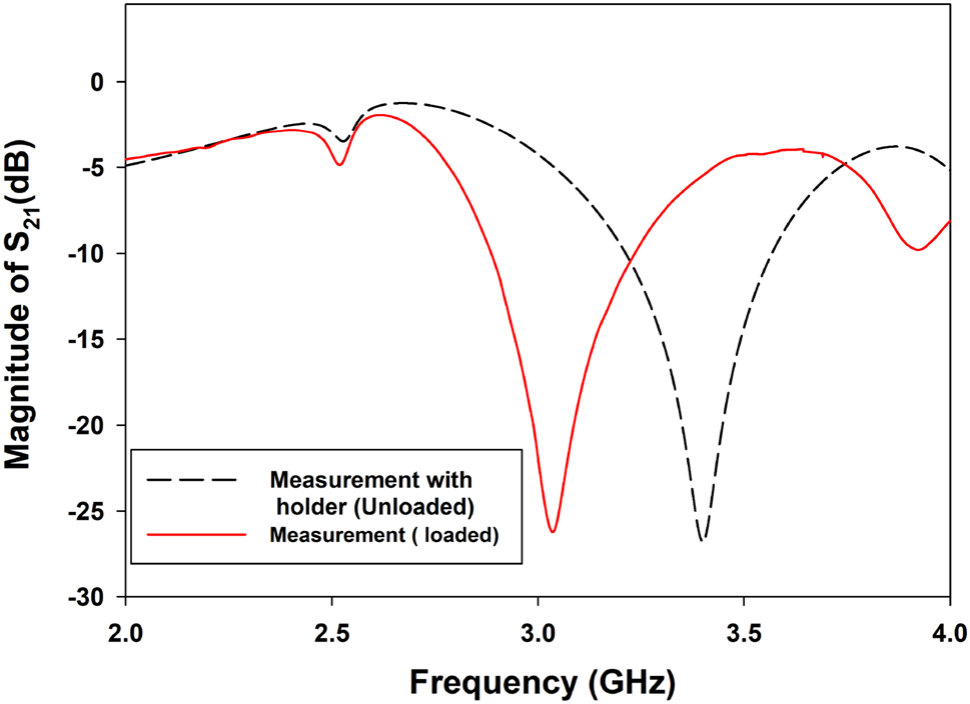
Measured result (solid line) of the loaded sensor with holder. The dashed line presents the measured results of the unloaded sensor with the holder.

The initial test is performed under a loaded sensor with a 1000 mg paracetamol tablet at room temperature (27 $^\circ$C). The tablet and the active zone of the sensor are not separated by any air gap. Figure 8 presents the experimental results of the loaded sensor. The measured transmission coefficient response is − 26.22 dB at a resonance frequency of $f_{ref}=3.0355$ GHz. The test clearly shows that there is a sensitivity to the presence of the paracetamol tablet with a frequency shift of approximately 444 MHz compared to the unloaded sensor response. This frequency shift is naturally due to the interaction of the radiated near field within the MUT, which is considered an additive dielectric substrate to the sensor. This obtained reference frequency $f_{ref}$ will be used to evaluate any frequency shifting when the loaded tablets are heated to different temperature levels.

**Figure 9:**
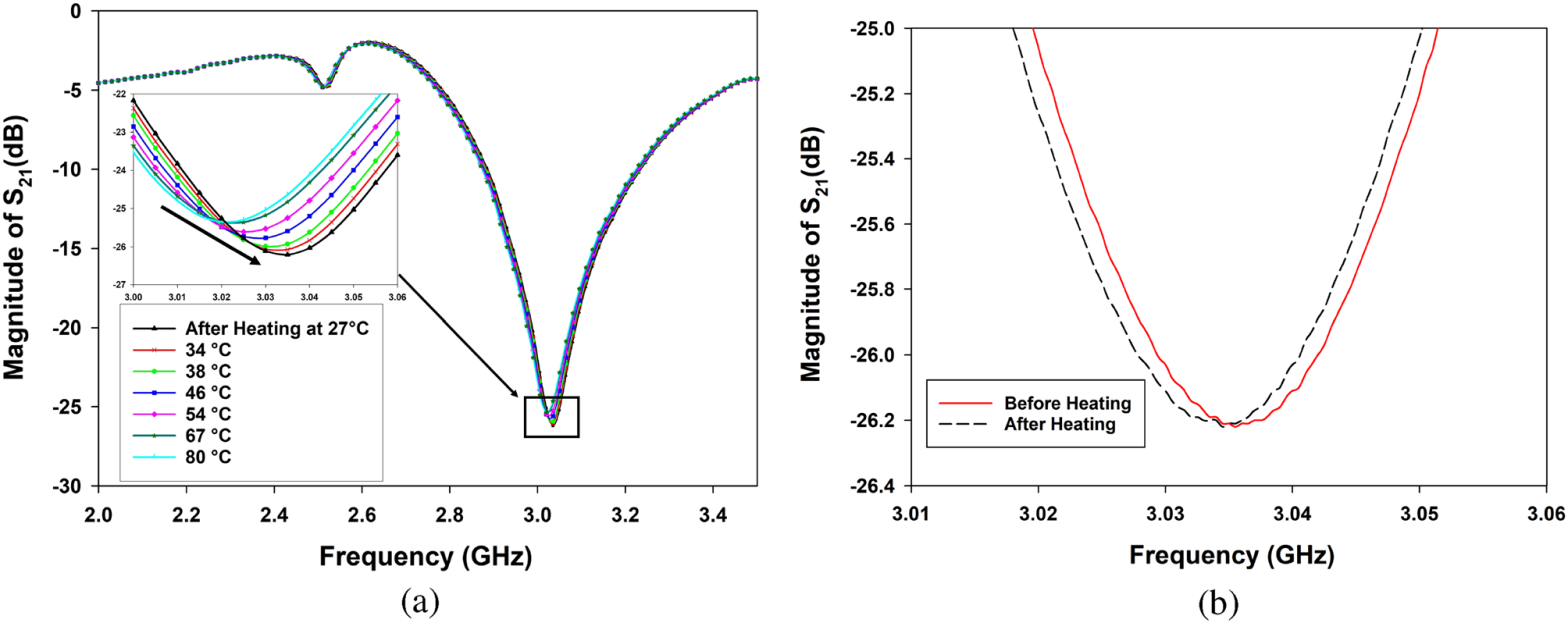
(**a**) Resonance frequency of the sensor versus magnitude of ${S_{21}}$, as temperature is decreased from 80 to 27 $^\circ$C. (**b**) Comparison between resonance frequency versus magnitude of ${S_{21}}$ before and after heating at 27 $^\circ$C.

In what follows, we study the correlation between different heating levels of the paracetamol tablets and their impact on the measured responses in terms of frequency shifting of the microwave sensor. After heating the S8 paracetamol sample at 80 $^\circ$C over 15 min, its temperature decreased back to room temperature (27 $^\circ$C). Every state’s resonance frequency as the temperature drops are measured versus its particular temperature value. The surface temperature of the drug was measured by a Medisana TM A77 thermometer. Figure 9a provides the resonance frequency response of the microwave sensor at different temperatures (80, 67, 54, 46, 38, 34, and 27 $^\circ$C, which is room temperature). Higher temperature values cause a decrease in the resonance frequency. Table 2 shows the resonance frequencies for each distinct temperature level. Figure 9b shows that after the tablet heating operation and stabilization of temperature at 27 $^\circ$C, which is considered the reference temperature, we note that the microwave response obtained by the sensor does not correspond to that taken before the heating operation at the same reference temperature. In fact, the resonance frequency drops to 3.0345 GHz, and then the corresponding microwave quality shifting is approximately 1 MHz. A positive $Q_{\mu W}$ value (in this case 1 MHz) distinctly serves as an indicative marker of potential temperature-driven variations within the pharmaceutical compound, consequently impacting its overall quality. This observed variation arises from the dynamic alteration of the compound’s dielectric properties in response to temperature changes, which in turn influences the dependence of its resonance frequency.

This phenomenon can be explained by the known heat effect caused by evaporating the tablet’s internal humid solvent. Higher temperatures could cause the creation of a differential pressure, which encourages the quick evacuation of any humid substance related to the internal water amount inside the tablet. In our context, this evaporation could decrease the effective dielectric permittivity of the heated tablet and consequently lower the resonance frequency of the sensor.

**Table 2:** Heating temperature versus resonance frequency (after heating).

Temperature $^\circ$C	Frequency of resonance = $f_{heated}$ (GHz)	$Q_{\mu W}$=$f_{ref}$-$f_{heated}$ (MHz)
80	3.0212	14.30
67	3.0235	12.00
54	3.0249	10.60
46	3.0260	9.50
38	3.0293	3.30
34	3.0322	3.30
27	3.0345	1.00

**Figure 10:**
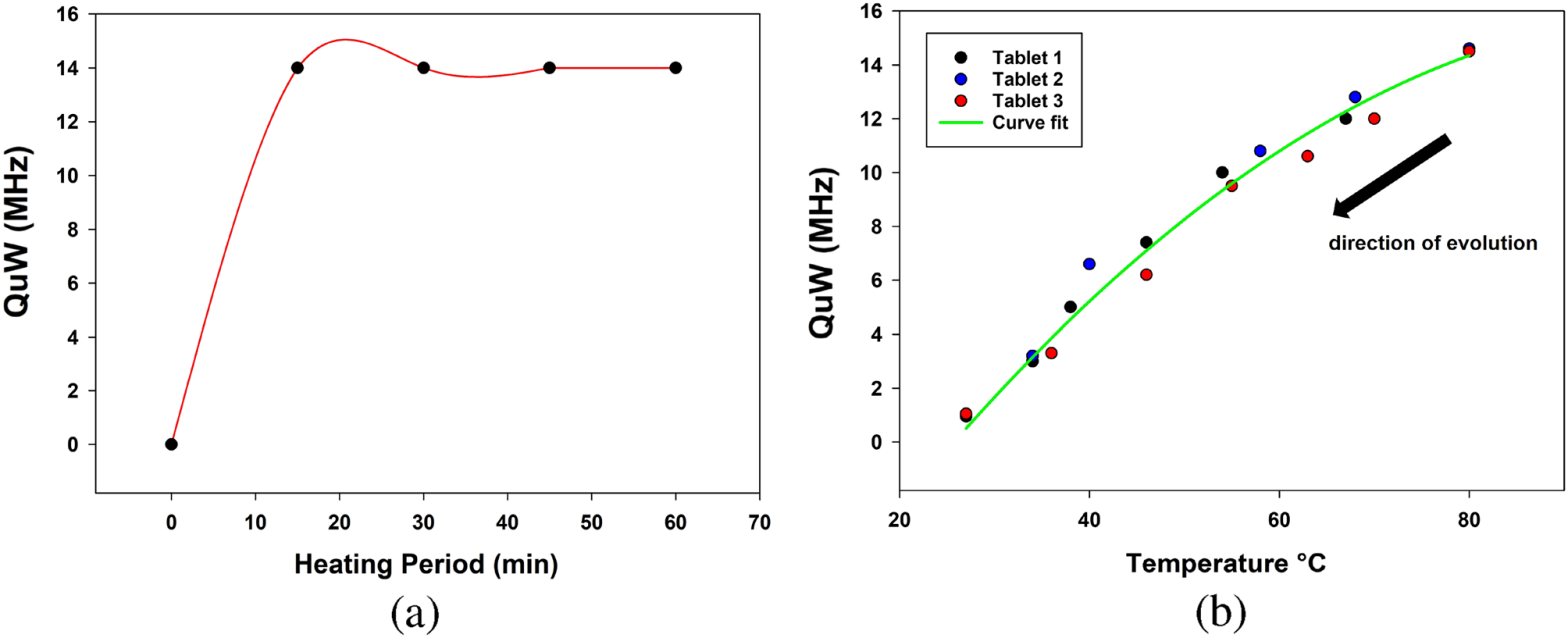
(**a**) Microwave quality shifting $Q_{\mu W}$ versus heating period at 80 $^\circ$C, (**b**) Average microwave quality shifting $Q_{\mu W}$ versus temperature for 3 different paracetamol tablets.

A set of four 1000 mg drug samples S1, S2, S3, and S4 were prepared and heated at 15 min, 30 min, 45 min, and 60 min, respectively, at a fixed temperature of 80 $^\circ$C. As shown in Fig. 10a, the $Q_{\mu W}$ becomes saturated after a 15 min heating period. As explained before, the heating procedure can cause evaporation of the internal moisture of the drug sample, but beyond 15 min, no additional amount of moisture evaporates. Thus, 15 min is the heating period used in future studies.

To evaluate the reproducibility of the sensor behavior, three identical 1000 mg paracetamol tablets were prepared and analyzed under the following procedure. S5, S6, and S7 were heated to 80 $^\circ$C for a 15 min period. Directly after reaching this heating period, the heated samples were carefully placed on the same sensing zone of the sensor. The $Q_{\mu W}$ was continuously measured during the tablet’s natural cooling, which itself is dependent on a room temperature of approximately 27 $^\circ$C. At each $Q_{\mu W}$ measured value, the corresponding temperature was recorded. Figure 10b shows the microwave quality shifting $Q_{\mu W}$ recorded from the three iterations using S5, S6, and S7 versus different heating temperatures. Each tablet’s temperature is lowered during the cooling process from 80 $^\circ$C to the room temperature of 27 $^\circ$C. The fitting curve (2) presents a linear regression model that better describes the variation of the temperature as a function of microwave quality shifting $Q_{\mu W}$ for each used sample. The exposure of drugs to high temperatures while being stored or transported could reduce their efficacy, and most licenses specify storage at 27 $^\circ$C or less. Knowing the instantaneous recorded value of $Q_{\mu W}$ versus temperature would allow us to identify all of the temperatures to which the drug was subjected while being transported.

In practice, it is critical to optimize the parameters of a predictive model using a variety of performance metrics. The root-mean-squared error (RMSE) is then determined from the linear regression model and the collected data. Additionally, the RMSE calculation was employed to examine the effect of changing the drug position effect in the measurement. 2.1 $^\circ$C of RMSE appeared and it could be due to the cooling ratio that can differ from each drug as well as the microwave measurement errors. However, the three iterations demonstrate that an identical $Q_{\mu W}$ value of roughly 14.3 MHz was obtained at the maximum heating temperature of 80 $^\circ$C.

2 $$\begin{aligned} T= 26.108*Q_{\mu W}^{2} + 1.438*Q_{\mu W} + 0.154 \quad (\ ^{\circ }C) \end{aligned}$$

3 $$\begin{aligned} RMSE= \sqrt{\frac{1}{4}*\sum _{i=1}^{k} (T_{i}-T)} \end{aligned}$$

Along with linear regression, several other models of machine learning were used in this study to calculate and compare various RMSE values. Ridge regression^36^ is a linear regression model with a quadratic constraint on the coefficients. A random forest regressor^37^ is an algorithm built from a collection of decision trees. It is simple to grasp, quick to learn, and yields outcomes that are transferable. A Huber regression^38^ is a type of linear regression used in robust regression that is less sensitive to data outliers than squared error loss. Table 3 provides an overview of the RMSEs of each method, demonstrating that the random forest regression and linear regression perform best by approximately 2 $^\circ$C of RMSE and are the appropriate models to correct such errors.

**Table 3:** RMSE value for each proposed model.

Model	Linear regression	Ridge regression	Random forest	Huber regression
RMSE	2.1085	3.0529	1.9223	3.0585

### Heating temperature

**Figure 11:**
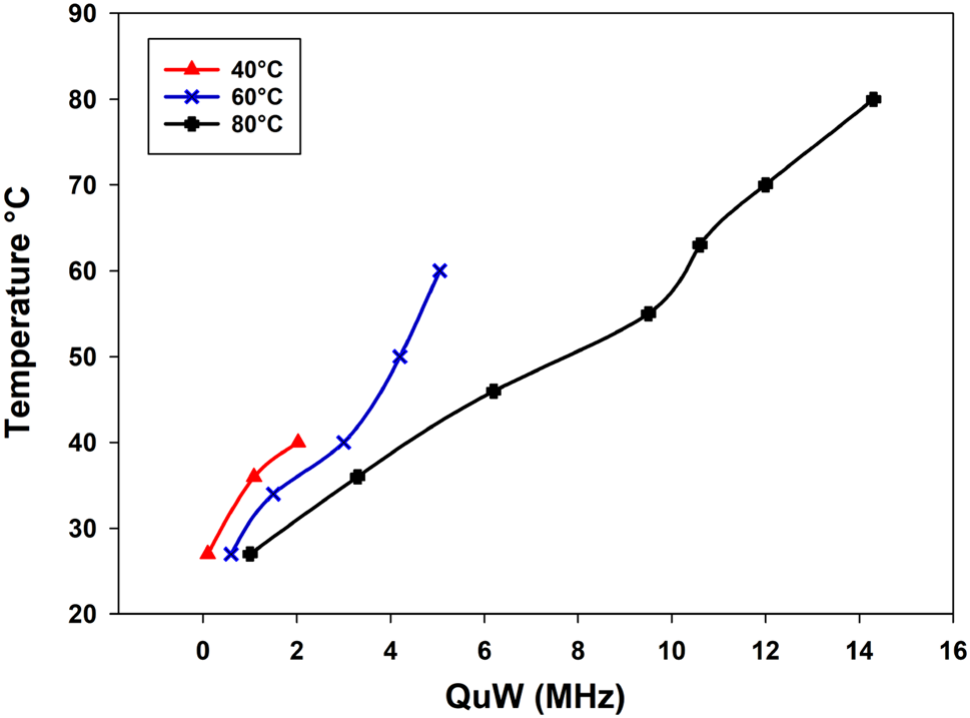
Comparison of different heating temperature (80 $^\circ$C, 60 $^\circ$C and 40 $^\circ$C).

In this experiment, we compared different heating temperatures (80 $^\circ$C, 60 $^\circ$C, and 40 $^\circ$C) for 15 min, as shown in Fig. 11. Heating a drug at a high temperature leads to a higher shift frequency. At 80 $^\circ$C, 14.3 MHz of microwave quality shifting $Q_{\mu W}$ is achieved. Therefore, after having cooled the sample, the resonance frequency of the sensor arrives at 1 MHz shifted to the reference. In contrast, 60 $^\circ$C and 40 $^\circ$C reach a shift frequency compared to the reference of 300 kHz and 50 kHz, respectively.

As a result, the change in water amount brought on by temperature vaporization shifts the resonance frequency of the loaded sensor, thus changing the dielectric characteristics of the sample. As a consequence, the rate of paracetamol variation (concentration) increased as the temperature increased. Moreover, for each of the three-sample situations, the suggested microwave sensor readings are successful in discriminating between different temperature levels.

To confirm the results obtained by the developed sensor, the LIBS technique was employed to examine the presence of C, H, N and O under different temperature conditions. The ingredients were extensively analyzed to ensure that they could be identified with high accuracy. To examine the spectral lines associated with different elements according to their wavelength and energy, the data were gathered using a laser source and spectrometer. The initial sample under test was a standard 1000 mg paracetamol tablet kept under the same conditions regarding the temperature (room temperature) and humidity. The spectra generated by LIBS were used to identify the substances contained in this medication. Regardless of the laser’s direction, the emission spectra were captured using a spectrometer positioned at a 90$^\circ$ angle to the sample’s surface.

**Table 4:** Peaks and their corresponding atomic elements (NIST DATABASE).

Element	Wavelength (nm)
Carbon	247.85
Hydrogen	656.27
Nitrogen (N1, N2)	742.36, 744.23
Oxygen	777.53

**Figure 12:**
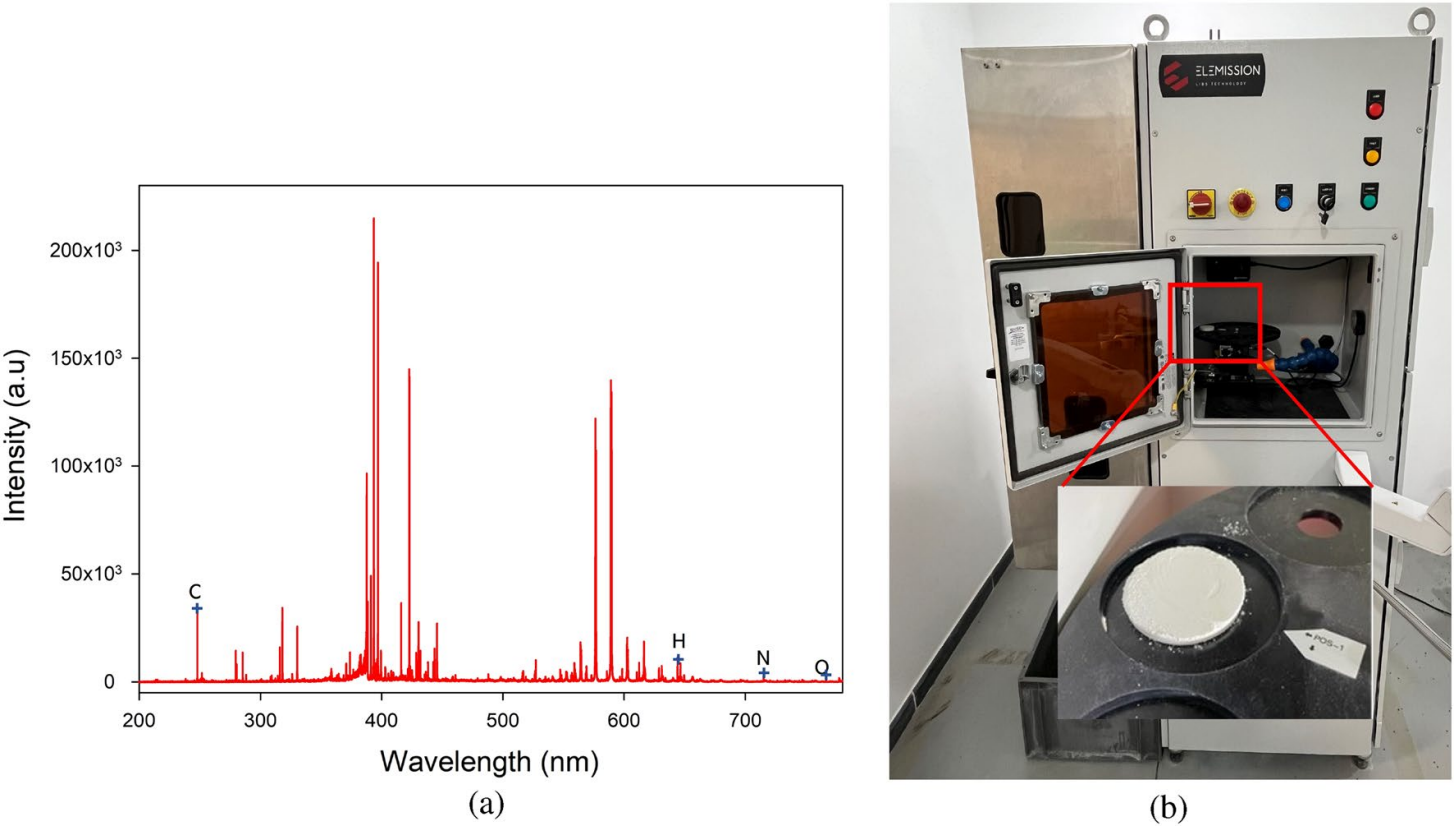
(**a**) LIBS spectra of the samples used in the study. (**b**) Drug location in LIBS.

**Figure 13:**
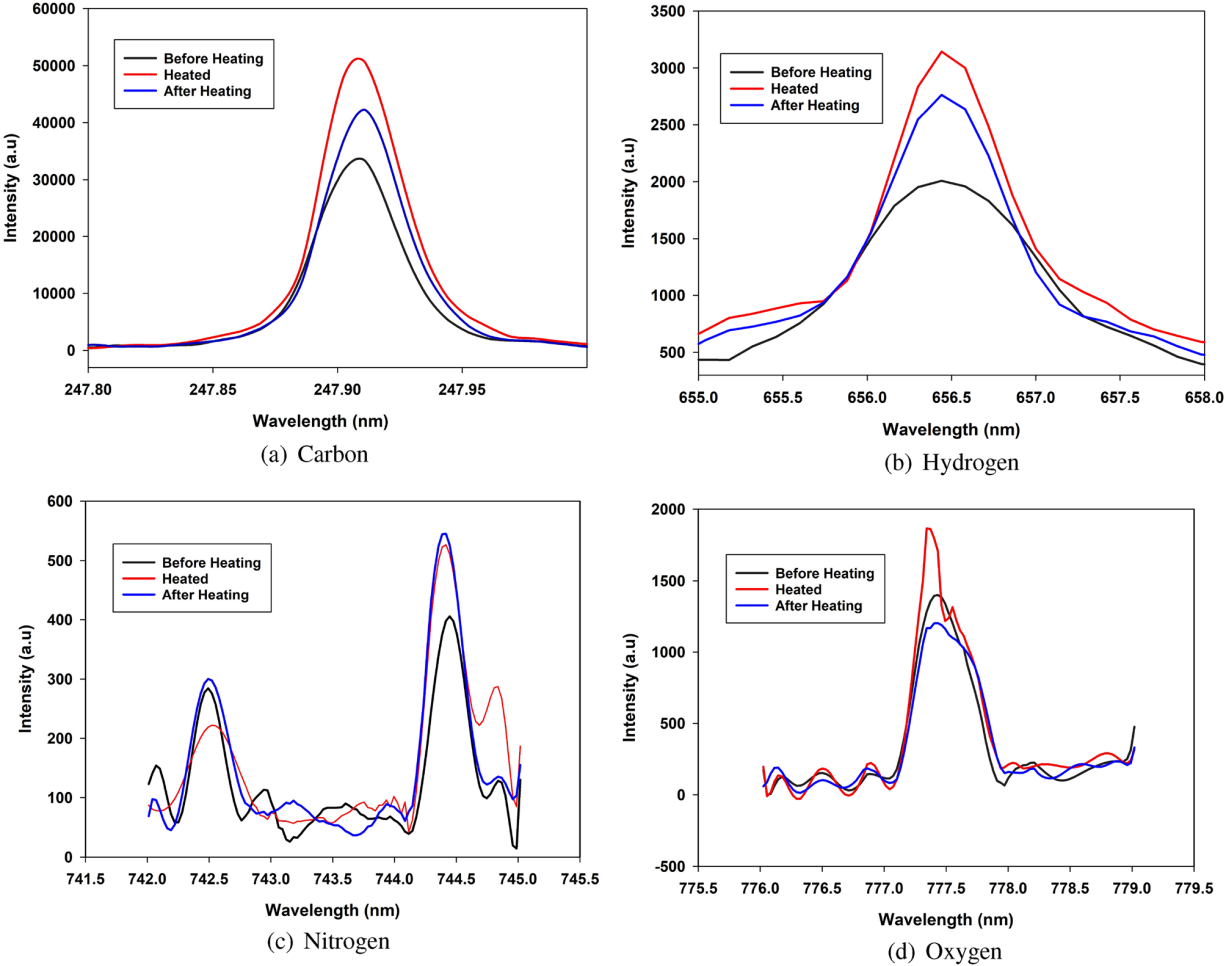
Different peaks for each detected element.

Figure 12a shows the spectra produced by LIBS to identify the elements present in a sample containing active agents and additional elements as inactive agents. This is the first investigation of such a sample. The identified elements are crucial in understanding the composition of the drugs. Furthermore, as shown in Fig. 12b, the sample was located in the LIBS instrument during the investigation. Fig. 13 shows a typical spectrum for each element present in the samples, displayed individually for the sake of exposition. Peaks belonging to nitrogen, oxygen, hydrogen, and carbon could be seen in every spectrum. These lines can be linked to the main constituents (active medicinal substances) of the drug, which are organic compounds. We believe that the additional metals might be impurities or sodium-containing excipients. The findings of the required constituents of the paracetamol samples are properly matched to the results from the NIST database in Table 4, compared to the observed peaks with LIBS in Fig. 11a. The chemical formula of paracetamol is C8H9NO2, which means that the molecule is composed of 8 carbon atoms associated with 9 hydrogen atoms, 1 nitrogen atom, and 2 oxygen atoms. The same procedure as the microwave measurement was applied to characterize the drug at different temperature levels. We heated the drug at 80 $^\circ$C for 15 min.

Figure 13 shows different peak levels for the three different temperature conditions. The black line indicates the peak corresponding to the drug at a room temperature of 27 $^\circ$C (before heating). The red and blue lines refer to a heated drug at 80 $^\circ$C and the drug after heating while it returns to 27  $^\circ$C, respectively. Figure 13 illustrates the changes in the atomic emission line intensities for carbon, hydrogen, nitrogen (N1, N2), and oxygen upon varying the heating temperature.

The parameters presented below are defined to qualitatively analyze the performance of the LIBS instrument. The intensity quality shifting $Q_i$ is expressed as:

4 $$\begin{aligned} Q_{i}=I_{ref}-I_{heated} \qquad (MHz) \end{aligned}$$

where $I_{ref}$ is the measured intensity of paracetamol at room temperature before heating. This intensity is considered as the reference. $I_{heated}$ is the measured intensity of the drug heated at 80 $^\circ$C.

It is notable from the values shown in Table 5 taken from peaks that carbon and hydrogen have large $Q_i$ values of 8884 and 812, respectively, between the intensities of the normal drug (before heating) and after heating at 80 $^\circ$C, unlike nitrogen (N1, N2) and oxygen, which provide small $Q_i$ values of approximately 16/140 and 198, respectively. Additionally, paracetamol consists of 8 atoms of carbon and 9 atoms of hydrogen, which are the most common elements in the structure. Thus, we consider carbon and hydrogen as the representative elements to characterize the phenomena in this work. The measured values shown in Fig. 12 represent the average of three repetitions; therefore, the risk of random fluctuations is small.

**Table 5:** Intensities for peaks of each elements in different condition).

ElementsConditions	Before heating 27$^\circ$C	Heated 80$^\circ$C	After heating 27$^\circ$C	$Q_i$
Carbon	33,396	50,673	42,280	8884
Hydrogen	1952	3144	2764	812
Nitrogen (N1/N2)	284/405	222/526	300/545	16/140
Oxygen	1400	1866	1202	198

**Figure 14:**
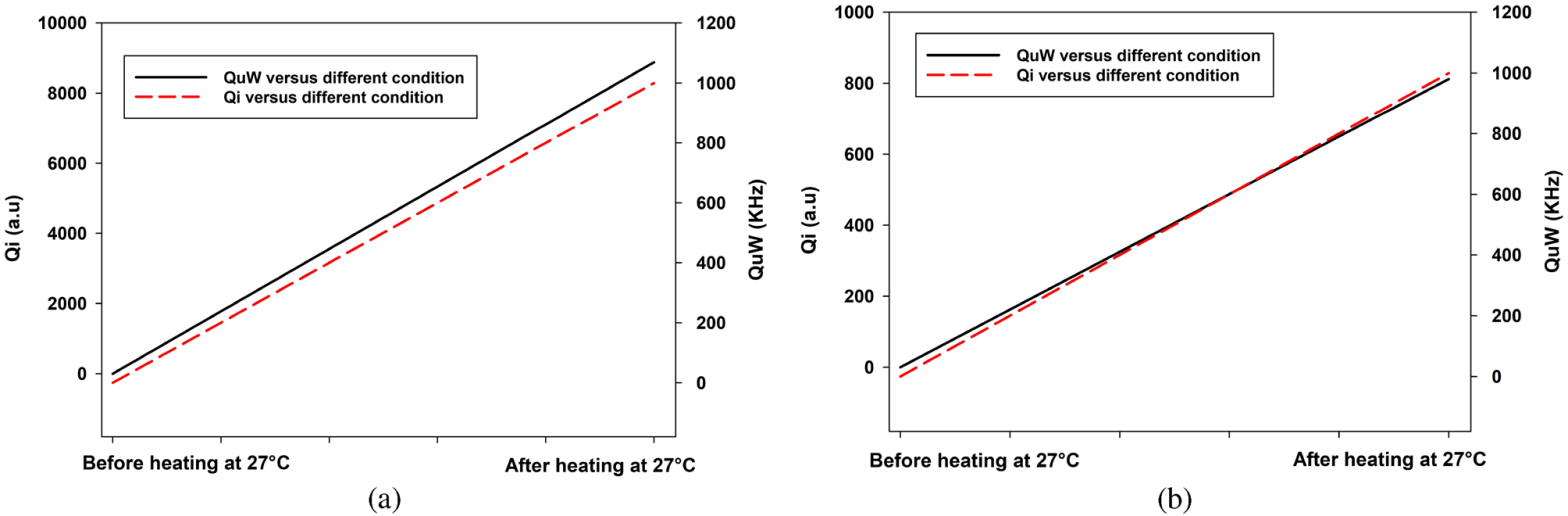
Comparison between $Q_{\mu W}$ and $Q_i$ for (a) Carbon (b) hydrogen.

It is clear in Fig. 14a,b that changes in temperature heating, which modifies the structure and dipole moment of water, definitely have an impact on the intensity of atomic emission lines from carbon and hydrogen. Increased heat gradients may create significant electrostatic fields that change the energy radiated by the sample, leading to an alteration in intensity level. This is due to the polarization of water induced by temperature fluctuations, which is an out-of-equilibrium phenomenon. The maximum intensity of both elements is observed during the measurement of the drug under heating at 80 $^\circ$C (red line), whereas the electric field induced by the temperature gradient should be larger. Otherwise, the intensity of the drug after heating (blue line) decreased because of the drop in temperature.

## Conclusion

In this paper, an SRR-loaded microwave planar sensor is proposed for tracking drug quality under different heat treatment conditions. A triple hexagonal split ring resonator (TH-SRR) is loaded in a circular patch, which is the sensing area in our system. The evaluation of sensor measurements centers around the microwave quality shifting ($Q_{\mu W}$), a parameter indicating how microwaves change behavior with shifting temperatures. This shift serves as an indicator of potential variations in the drug’s properties. By analyzing these shifts, we indirectly assess if the drug has been affected by temperature-induced changes, providing insights into its stability and potential damage The TH-SRR-based sensor has the benefit of a very strong and confined intensity between the resonator edges, which allows for a good interpretation of the smallest variations in drug properties caused by different heating conditions from 40 to 80 $^\circ$C. By locating the drug in this region and evaluating the change in the frequency response of the sensor, the microwave quality shifting $Q_{\mu W}$ is determined. In particular, heating a drug at 80 $^\circ$C has shown a noticeable $Q_{\mu W}$ of approximately 1 MHz. The sensor exhibits $Q_{\mu W}$s of 300 kHz and 50 kHz at 60 $^\circ$C and 40 $^\circ$C, respectively. Additionally, a comparison study is conducted using an analytical technique for determining the elemental composition of the drug. It is shown that LIBS presents an outstanding intensity quality shift of 8884 and 812 for carbon and hydrogen, respectively. Moreover, it demonstrates good agreement with the microwave quality shifting $Q_{\mu W}$. The findings of this study could be regarded as a model shift in microwave sensing for customized pharmaceutical applications such as monitoring the quality of drugs, which opens the door for their commercial exploitation.

## Data Availability

The datasets generated and/or analyzed during the current study are available in the OneDrive Cloud repository, you can access all the data through this link https://um6p-my.sharepoint.com/:f:/g/personal/youness_zaarour_um6p_ma/EshQOEITZh9JoT8e9DhsPuMB8mlh8soj82SRejNNC7C4mQ?e=rfa61P.
